# Improving TB Surveillance and Patients' Quality of Care Through Improved Data Collection in Angola: Development of an Electronic Medical Record System in Two Health Facilities of Luanda

**DOI:** 10.3389/fpubh.2022.745928

**Published:** 2022-03-24

**Authors:** Claudia Robbiati, Maria Elena Tosti, Giampaolo Mezzabotta, Francesca Dal Maso, Ofélia M. Lulua Sachicola, Paulo Siene Tienabe, Joseph Nsuka, Marco Simonelli, Maria Grazia Dente, Giovanni Putoto

**Affiliations:** ^1^Doctors With Africa CUAMM, Padova, Italy; ^2^National Center for Global Health, National Institute of Health (ISS), Rome, Italy; ^3^Independent Researcher, Bene Vagienna, Italy; ^4^Ministry of Health of Angola, Luanda, Angola

**Keywords:** TB, surveillance, Angola, Electronic Medical Record (EMR), quality

## Abstract

TB Programs should promote the use of digital health platforms, like Electronic Medical Records (EMR) to collect patients' information, thus reducing data incompleteness and low accuracy and eventually improving patients' care. Nevertheless, the potential of digital health systems remains largely unexploited in low-resource settings. Angola is one of the 14 countries with a triple burden of TB, TB/HIV and MDR-TB (multidrug-resistant TB) and it is among the three countries, together with Congo and Liberia that have never completed a drug-resistance survey so far. The Sanatorium Hospital of Luanda and the Tuberculosis Dispensary of Luanda are the two reference health facilities in Luanda dealing with most of the TB cases, and they both rely entirely on paper-based data collection. The aim of this paper is to describe a three-stage process for the development of a TB EMR system in these two health facilities of Luanda and to share the lessons learned. The description is focused on the activities that took place from March 2019 to January 2020. Main lessons learned were identified in the importance of engaging all the stakeholders in the development process, in the mainstream of the “think digital” transition, in the promotion of a monitoring and evaluation (M&E) culture and in the planning of the system's sustainability. This approach may be replicated in similar contexts where the development of a TB EMR system is sought, and the lessons learned could assist and facilitate the programming of the interventions.

## Introduction

The Tuberculosis (TB) pandemic, including its multidrug-resistant (MDR-TB) form, is a global health threaten. TB is one of the top 10 causes of death worldwide and the leading cause of death from a single infectious agent. An estimated 10 million people fell ill with TB in 2019 and 1.2 million people died (1).

The WHO End TB Strategy aims to end the global TB epidemic, with targets to reduce TB deaths by 95% and incidence by 90% between 2015 and 2035, and to ensure that no family is burdened with catastrophic expenses due to TB (2). In the years 2015–2019, TB incidence rate dropped by 9%, however this was less than halfway to reach the End TB Strategy milestone of 20% incidence reduction between 2015 and 2020 (3).

To achieve the End TB strategy goals and improve patients' management, effective surveillance systems are needed to measure and monitor the burden and the determinants of the disease. Implementing a functional TB surveillance system often remains challenging in many low- and middle-income countries (4). TB programs should promote the use of digital health platforms to collect patients' information, to reduce time and efforts to fill paper forms and to avoid common mistakes like data incompleteness and low accuracy. Digital health interventions can strengthen surveillance and monitoring, program management and ultimately patients' care (5).

Introducing a new digital health system needs an enabling environment and to deploy a formative stage to document bad and good practices, pivotal for the uptake of the new system (6). Therefore, a comprehensive approach is required, that starts with gaps and needs identification and the description of the patient's pathway for TB care (7).

Angola is among the 14 high burden countries for TB, TB/HIV and MDR-TB prevalence and is among the three countries, together with Congo and Liberia, that have never completed a drug-resistance survey so far (1). Moreover, according to the WHO country profile, Angola had a treatment success rate of 25% in 2017, much lower as compared to a pooled rate of 76.2% in sub-Saharan Africa countries in the last decade, according to a recent systematic review (8).

The Angola TB network is made of Sanatoriums, TB Dispensaries (DAT), Diagnostic and Treatment Units (UDT with smear laboratory), and Treatment Units (UT without smear laboratory). The TB surveillance system in Angola is weak, with main gaps regarding cases notification, data reporting and elaboration, and quality of data collected (9). The Angolan TB National Program (PNCT) uses a digital database to collect aggregated data from the network, however countrywide TB facilities continue to rely on paper-based tools leading to incompleteness, poor accuracy of data and delays in reporting. Therefore, it is imperative to enhance data collection and management in order to strengthen TB surveillance, programmatic decisions and patient's management. To this purpose, the implementation of user-friendly Electronic Medical Record (EMR) systems could improve data quality and strengthen the continuum of care by ensuring adherence to clinical guidelines and reducing errors in data recording and reporting. A study based in Malawi documented the positive outcomes of a point-of-care EMR system for HIV-TB patients in a public clinic (10). In Rwanda the use of EMR data for evidence-based clinical decisions improved HIV patients monitoring (11). A study in Kenya reported significant improvements in data quality through the implementation of a cloud-based EMR system for maternal and child health (12).

A systematic review showed that the factors that challenged the widespread use of EMR systems in Sub-Saharan Africa were the high costs of procurement and maintenance of the EMR system, poor implementation planning, issues with electricity supply and internet connectivity, and user's limited computer skills (13). Strategies such as phased implementation planning, financial sustainability, appropriate EMR system and training of users, showed positive results in EMR systems implementation.

## Context

Luanda is the capital city of Angola with one-third of the Angolan population living in conditions of overcrowding and poor sanitation. Luanda has the highest TB mortality rate in the country (13.9^*^100.000 inhabitants) and 245 MDR-TB cases over 534 (46%) notified at country level in 2018 (14). A recent study also showed that in Luanda chronic diseases like diabetes are an important comorbidity for TB patients (15).

The Sanatorium Hospital of Luanda (HSL) and the Anti-Tuberculosis Dispensary (DAT) of Luanda are the two reference health facilities in Luanda dealing with most of the TB cases, and being also reference centers for the diagnosis and management of MDR-TB (HSL) and provincial reference center for the PNCT (DAT). These two health facilities collect clinical and surveillance data entirely on paper medical records and registries and this may result in poor quality of data and ultimately poor patients' care.

The project “Stop TB and TB/HIV in Angola: Improving Access to TB and HIV treatment by enhancing diagnostic quality and patient management in the Province of Luanda” funded by the Italian Cooperation Agency (AICS—Agenzia Italiana per la Cooperazione allo Sviluppo) and implemented by the Italian non-governmental organization “Doctors with Africa CUAMM” with the technical support of the “Italian National Institute of Health” (ISS) aimed at improving the quality of diagnosis and management of TB and TB/HIV patients at the HSL and the DAT of Luanda. The project, among other activities, included the development of a TB EMR system, based on an enhanced TB medical record and the setting up of software and hardware features, to be piloted in the two health facilities. The EMR system development was a three-stage process and included first a situation analysis, second the development of an enhanced medical record and finally the development of the EMR system. The situation analysis supported the identification of the patients' flow and actual gaps and needs in data recording and reporting at the HSL and the DAT of Luanda. The results of the situation analysis were used to develop an enhanced medical record that aimed at filling the gaps and needs identified, and finally in the development of the EMR system. The process was consolidated through a workshop for the staff of the two health facilities (around 40 people) involved in the data collection with the double aim of promoting awareness about the importance of data quality and getting acquainted with the TB EMR system.

The aim of this paper is to describe the three-stage process for the development of the EMR system and share the lessons learned. The description is focused on the activities that took place from March 2019 to January 2020.

The process could be replicated in other health facilities in Angola and other similar contexts to improve TB surveillance, control and quality of care, to boost the achievement of the WHO END TB strategy.

## Stage 1: Lay the Foundation for the EMR System Development

### Stakeholders' Engagement

A series of meetings, interviews, site visits and observations were arranged involving local stakeholders in order to engage them in the design of the EMR system and to promote ownership and sustainability of the system. Representatives of the Ministry of Health and local authorities, key staff of the two health facilities, health care workers (HCWs), international organizations and other local NGOs with similar experiences were involved in this step, to gain their insights and recommendations, discuss perceived barriers and come to a consensus on a plan of actions for the implementation of the EMR system and ensure its adherence to the national guidelines. The process involved around 20 stakeholders that were constantly consulted along the duration of the project.

### Situation Analysis

From March to September 2019 the EMR expert team, including two epidemiologists from the Italian National Institute of Health, CUAMM project manager, a TB specialist consultant and a software developer consultant, conducted specific site visits at the two health facilities to gain full knowledge and understanding of the patients' flow and data collection system. The final aim was to design a user-friendly EMR system that could meet the stakeholders needs, while improving data collection and reporting. During the visits, the TB patients' flow was analyzed from the entrance to the discharge and the data collection points, established by the health facilities in accordance with the patient's assistance and care pathway, were identified and described, including the data collection tools in use.

Interviews with key staff and HCWs at the two health facilities were also carried out to feed their suggestions into the development of the EMR system. A purposive sample of data was collected and analyzed with the aim of assessing the quality of the information collected.

The situation analysis allowed to identify strengths and weaknesses of the current data collection system in the two health facilities and to define areas of improvement. Moreover, this stage identified opportunities and threats to the implementation of the EMR system.

Strengths, weaknesses, opportunities, and threats (SWOT) that resulted from the situation analysis are reported in Table 1.

**Table 1:** SWOT analysis.

**Strengths**	**Weaknesses (areas of improvement)**
Key stakeholders' commitment to the EMR system development and implementation; Previous similar experiences to benefit from; Presence of local technical expertise to draw upon.	Suboptimal data collection and management, and ultimately patients' care; Manual for clinical and programmatic management of TB and MDR-TB only available as an electronic draft, and not widely distributed (at the time of the study); Gaps and delays in the reporting process; Poor data/information quality; No M&E functioning system.
**Opportunities**	**Threats**
Window for capacity building initiatives; Reducing workload of HCWs related to paper-based data collection; Strengthening national surveillance system; EMR system could be scaled up in other health facilities and for community follow-up.	Disturbance of daily activities during the system roll-out; HCWs poor digital skills; HCWs high workload; System uptake resistance; Financial sustainability.

### Areas of Improvement (Weaknesses)

Data collection and reporting presented some gaps and inaccuracies, mostly linked to the great flow of data to be collected on paper-based tools, which could also undermine data privacy and security. Main areas of improvement were identified in the use of multiple forms and codes for the same patient that could lead to misinterpretation of data, including patients' identification, or loss of information. In addition, the need to improve the timeliness and accuracy of internal and external reports that rely only on paper-based registers emerged as an important issue, to support programmatic decision-making and TB surveillance. Finally, some cruxes along the pathway of patient's care do not provide for the collection of data, thus some essential information for patients' management is lost.

An adequate M&E system should be implemented, and data/information quality should be enhanced, particularly regarding the quality of patient's information recorded (eg., completeness, readability, numeration etc.,); quality of clinical management of patients' information (eg., bacilloscopy results/timing, HIV test result, body weight etc.,); quality of data flow information (eg., timing of internal reports, coherence between registries and reports, reports availability etc.,). As an example, patients that do not perform bacilloscopy (BK) are often reported as BK negative, while they should be reported as not assessed. Reported cases of relapses are very few, raising the question that relapses might be often treated as new cases without ascertaining the possible development of a resistant form of TB. The final treatment outcome is most of the time absent in the registries, possibly linked to the poor patient's follow-up.

At the time of visiting the facilities there was no updated TB manual providing consolidated guidelines about recording and reporting procedures, which might at least partially explain the occurrence of the shortcomings noticed above. The same can be said for the management of MDR-TB.

The areas of improvement identified suggested the need to enhance the medical record currently in use and refine the current data collection and reporting system, to improve patients' management and quality of care (Table 2).

**Table 2:** Examples of indicators of weaknesses in the current data collection system and the correspondent EMR system response.

**Weakness (areas for improvement)**	**EMR system response**
Lack of registration of the patients dismissed after the first visit because not considered suspect TB cases. The whole facility workload is therefore missing.	All the patients are registered at their 1^st^ access to the health facility.
Use of multiple forms and codes for the same patient.	Univocal code, univocal record saved indefinitely and accessible from each established data collection point.
Delays and incompleteness of internal and external reports.	Specific reporting feature of the system to ensure delivering of internal and external reports in accordance with the requirements of the national reporting system.
Poor or inexistent data collection system at some cruxes of the patient's care pathway.	All the needed data collection points of the patient's care pathway are included as active units of the EMR system.
Data quality.	Guided choice, mandatory answers, improved M&E system.

## Stage 2: Enhancing the TB Medical Record

After discussing the gaps and needs of the current data collection system emerged during the situation analysis, the EMR expert team fed the results in the elaboration of an enhanced TB medical record. To this purpose, the two medical records and patients' forms in use at the two health facilities were used as a starting point. The DAT used the standard clinical record adopted by the National TB Program with essential information about diagnosis and treatment. The HSL medical record was based on the National TB Program standard record, but included more information, reflecting its role in the management of complex cases and MDR-TB cases.

A preliminary version of the enhanced TB medical record was discussed with key stakeholders to assess information clarity, content and sequencing, and compliance to national guidelines, and adjusted accordingly.

A formative workshop was arranged in January 2020 in the two health facilities to introduce the HCWs (around 40) to the enhanced medical record and to instruct them about their use for the digitalised data collection. The formative workshop allowed the HCWs to increase their awareness about the importance of data collection for TB surveillance, for the quality of the patient management and to enhance the accuracy of data reported to the National TB Programme for programmatic decisions.

The enhanced medical record is divided into several sections that follow the patient's clinical pathway (Table 3).

**Table 3:** Enhanced TB medical record sections description.

**Medical record sections**	**Section description**
Patient registration and socio-demographic information.	All the patients are registered, also patients without clear TB symptoms. The registration also includes the attribution of a univocal patient number that will follow him/her throughout the clinical course. Socio-demographics data are collected.
Patient classification and clinical information.	Patient classification (new, retreatment, transferred) and related information is recorded, together with main TB symptoms, signs, risk factors (contact with a TB case, MDR-TB cases among the relatives, HIV infection and therapy, diabetes, pregnancy, smoke, alcohol) and body weight.
First medical consultation outcome.	Based on the clinical assessment and the evaluation of risk factors during the first visit, patients could be defined as ≪suspect TB case≫ and referred for diagnostic confirmation or hospitalization. If the patient is not a TB suspect, the diagnosis and therapy are reported in the medical record, that is subsequently closed.
Diagnostic information (laboratory and radiology).	Results of bacilloscopy, GeneXpert, culture, antibiogram, RX, biopsy, HIV test, clinical biochemistry are reported. For the bacilloscopy, reasons for not performing the test need to be specified.
Case classification.	Following the diagnostic process, the TB diagnosis is confirmed or excluded. The TB case is further classified according to the pulmonary or extrapulmonary location and to the presence of HIV co-infection. Contacts of people living with the patient are recorded, to promote active case finding.
Treatment plan.	Type of treatment according to the national guidelines, dosage, starting and end date and observations are recorded.
Therapy follow-up.	Date, type and quantity of medications delivered to the patient along the course of treatment are recorded. Also, patient's eventual delay and reason, and treatment compliance are recorded, in order to determine the next date for the delivery of medications (this will be automatically calculated in the EMR system).
Follow-up consultations.	During follow-up consultations (2, 5, 6 months for drug-sensitive TB and every month for 20 months for MDR-TB), therapy compliance, symptoms, side effects, weight monitoring and observations are recorded.
Diagnostic follow-up (laboratory and radiology).	Follow-up results of diagnostic tests (bacilloscopy, GeneXpert, culture, antibiogram, RX, biopsy, HIV test, clinical biochemistry) are recorded.
Treatment outcome.	Treatment outcomes according to national guidelines are reported.

## Stage 3: Setting up the EMR System

The EMR expert team fed the results of the situation analysis and the contents of the enhanced medical record in the design of the EMR system. Software and hardware components were defined, according to the stakeholders' needs and recommendations. The EMR system has been designed as a real-time, point-of-care, internet-based system based on open-source software that employs C# and Java language and the MSSQL server 2017. The system is available in Portuguese and English. Although the system is web based, and it allows to work online and offline, so far it has been configured to be used on a LAN (Intranet) at the health facility level in offline modality only.

The system is accessible only to authorized staff that has been provided with username and password and keeps an audit trail by tracking users, locations, and time for all the accesses. Data is encrypted during offsite data back up using secure encryption protocols. The system owns the technical features needed to communicate with the District Health Information Software 2 (DHIS2) (16), the Health Management Information System that is increasingly being used by the TB National Program for aggregate data reporting. The system can produce internal and external reports according to formats and indicators in use in the two health facilities and within the TB National Program. Multiple testing sessions assessed the usability of the EMR system.

**Figure 1:**
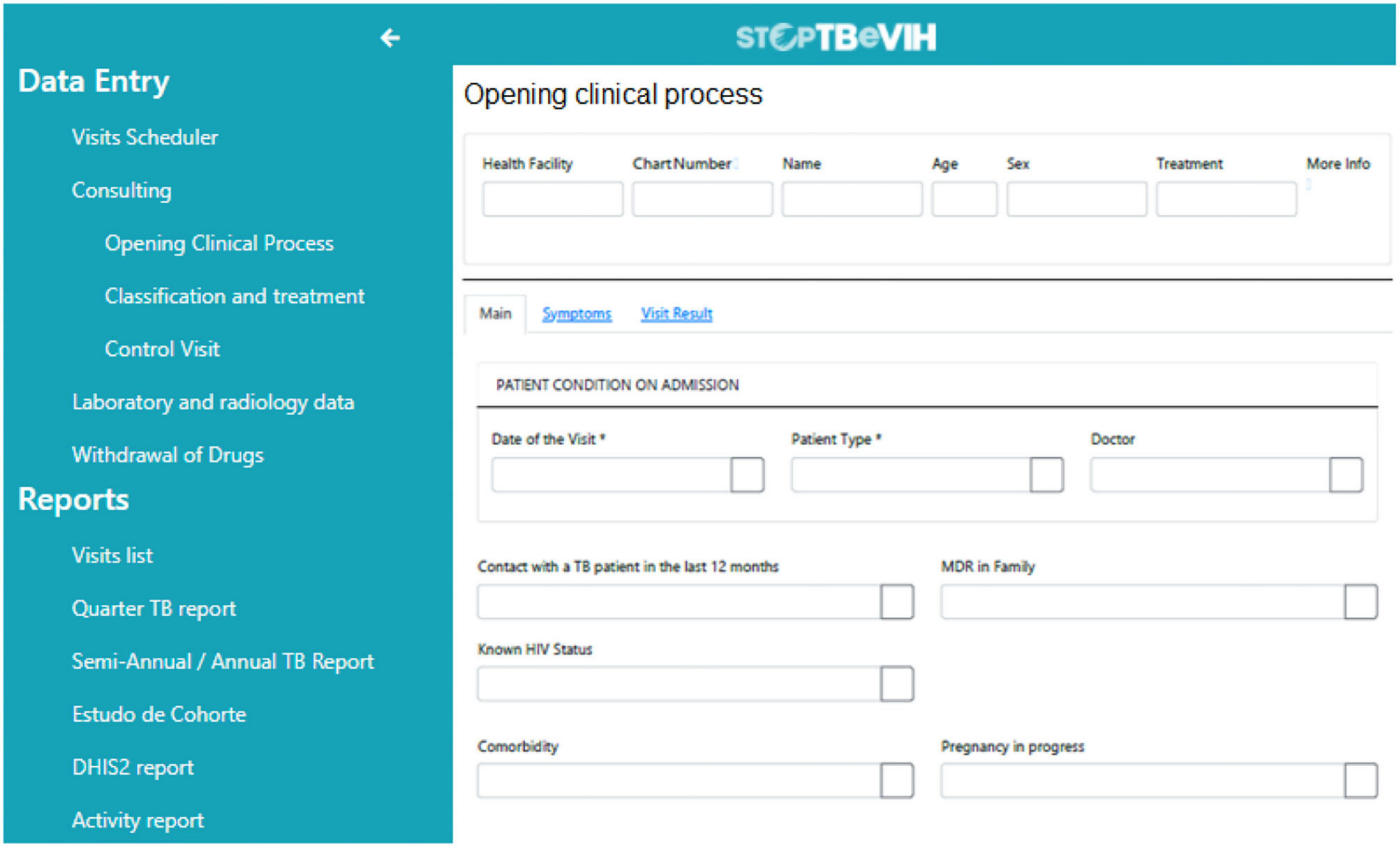
EMR system print screen.

A print screen of the EMR system is shown in Figure 1.

## Lessons Learned

The following lessons learned were collected during the project, through a continuous consultation process with the stakeholders involved.

### Engage the Stakeholders

Local stakeholders were identified as the representatives of local health authorities, key staff and HCWs of the two health facilities and other TB health facilities, representatives from international organizations and NGOs working in the same area. Engaging all the stakeholders in an inclusive process allowed to promote the ownership and buy-in of the final product and its sustainability, to encourage trust among all the actors, to share a common vision about how to overcome the challenges and to identify capacity building needs (10).

Introducing innovation in information systems needs to be supported by an enabling environment, since it could be perceived by the final users more as a challenge than an advantage, unless tangible benefits are clearly communicated (17). As an example, all the stakeholders agreed that the reporting process was time-consuming, resulting in delays and inaccuracies, therefore the new EMR system aimed at simplifying the process while improving the quality of the data reported. In addition, the stakeholders agreed to the fact that the time spent by the HCWs recording data on paper-based forms and registries, could have been better employed for the management of the patients and for improving the quality of care.

The stakeholders were engaged during the whole process and were consulted at each step, to consult with them about the best strategy to meet their needs. This allowed to enhance the medical record with the information they needed to improve the management of the patients. The patient socio-economic and clinical data were enriched, and even if this may increase the duration of the first visit, the information collected will enable a better care of the patient. The pilot of the system should take this aspect into consideration to find an operational balance.

### Mainstream the “Think Digital” Transition

Digital health is a cultural transformation, mostly in low- and middle-income countries, where technology is not yet fully exploited. Local stakeholders endorsed the digital transition and “think digital” promoters in the two health facilities were asked to endorse the digital health cause and to provide evidence of the benefits to the other HCWs (4). These promoters started to support HCWs of the two health facilities few months before the EMR system piloting, in order to guide them to a smooth transition to the digital system and highlight the gaps that the EMR system could fill.

Challenges to the “think digital” transition were mostly identified in the poor digital skills of the HCWs and resistance to the new system due to previous negative experiences. To address these challenges, the EMR system was developed as an easy-to-use system, with a simple interface to reduce complexity. Standard desktop computers with large screens were bought and a basic IT training has been planned at the beginning of the piloting phase to uniform digital skills of the HCWs. Continuous training on the job activities and a stepwise rollout of the EMR system during the piloting stage was planned to allow the HCWs to adapt to the transition from a paper-based system, without causing any disruption to the daily activities.

The development of the EMR system had to be gradual, to have all the stakeholders on board and feed all the recommendation in the process. The organizational leadership of the two health facilities supported the development of the system and addressed critical issues that arose during the process.

### Promotion of an M&E Culture

The situation analysis showed the importance of strengthening the monitoring and evaluation system and culture, particularly the quality and the regular evaluation of the data collected. Moreover, it highlighted the necessity to define roles and responsibilities for M&E activities. From the meetings with local stakeholders and HCWs of the two health facilities it was evident that awareness-raising activities about the importance of data quality for TB surveillance and patients' care, should be regularly performed.

Highlighting the practical advantages of the EMR system for data accuracy and timeliness during the recording and reporting to the National TB Program, was a strong argument that allowed to sow the seeds for an M&E and accountability culture to grow among the stakeholders. Another opportunity to promote M&E culture was to identify M&E champions among local stakeholders and in the two health facilities, to encourage their peers to effective data collection, monitoring and evaluation. The M&E champions were seen by the staff of the two health facilities as a valuable support to improve their M&E and data quality skills. Linking the concept of data quality to a better management of patients, to a reduced time in reporting and to an improved functionality of the facility allowed the HCWs to consider data quality as a priority. The M&E culture promotion will continue to be strengthen during the planned training on the job activities during the the piloting stage of the EMR system.

During the formative workshop in January 2020 in the two health facilities, HCWs were introduced to the importance of accuracy, transparency and accountability of data collected and reported to improve TB surveillance, programmatic decisions and clinical management of patients.

### Plan for Sustainability

Stakeholders' engagement was the first step toward the EMR system sustainability, in order for them to endorse the ownership and the buy in of the system (18). After the piloting and evaluation phases of the EMR system, the local stakeholders would need to endorse the system running cost that consist in the hardware and software maintenance and possibly in capacity building activities, like training on the job to increase uptake and utilization of the system by the HCWs.

The involvement of local software programmers in the development of the EMR system will allow system improvement and maintenance beyond the project, without the need to involve foreign consultants, and therefore decreasing the costs. As a matter of fact, looking for in-country expertise can promote initiatives that are more likely to be sustainable than those depending heavily on external support (4).

The possibility for the EMR system to be integrated with the DHIS-2 software, conferred to the EMR system the possibility to be extended to other health units in the country. Also, the flexible features of the system, the user-friendly interface and the easy programming language, will allow it to adapt to different needs and skills, therefore increasing its chance of scalability and sustainability.

## Limitations

The situation analysis was done only in two health facilities. Other relevant needs of other health facilities of the Angolan TB Network might not have been considered.

However, the enhanced medical record and the EMR system were developed considering comprehensive needs of all the stakeholders involved, related to the enhancement of data collection and management, in order to allow replicability in other TB services in Angola.

## Conclusion

This study was a first attempt to promote a digital health intervention in the context of the TB National Program in Angola. The study was a three-stage process. The first stage allowed the identification of gaps and needs in the data collection systems of the two health facilities. The second stage included the development of the enhanced medical record, based on the findings of the situation analysis. The third stage supported the development of the EMR system. The EMR system developed in the two health facilities in Luanda owns all the features to improve data collection, data quality and finally patients' management and could be exported to the other health facilities across the country.

The main lessons learned in the process were to engage all the involved stakeholders since the beginning and during all the stages; to promote the “think digital” cultural revolution; to support the development of an M&E system through all the steps of the data collection flow; to include sustainability of the system among the priorities areas to be discussed before starting the process. The lessons learned were similar to other studies in similar context (10), thus reinforcing their relevance.

Covid-19 pandemic caused severe disruption in the planned activities for the piloting of the EMR system in the two health facilities. Nevertheless, once the health emergency situation will decrease, the system will be piloted, evaluated and refined. According to other similar studies, during the piloting stage of the EMR system, particular care needs to be focused on enhancing the accuracy of the data recorded (19, 20). The evaluation of the EMR system will target the actual impact of the system on data quality and quality of care. Weaknesses related to data collection that were highlighted during the situation analysis, and the compliance to the EMR system requirements, will be assessed. This will also include an analysis on how well the EMR system matches the actual workflow of patients, and if this could be improved to enhance patient's care.

The TB EMR system would be an important tool to be scaled up to other TB services of the Angolan network, allowing to improve data collection and management and finally to strengthen national TB surveillance, programmatic decision-making and patients' quality of care.

## Data Availability Statement

The data were retrieved from patients' registries. Requests to access these datasets should be directed to c.robbiati@cuamm.org.

## Author Contributions

CR, MD, MT, and GM contributed to conception and study design, data acquisition, and interpretation of results and drafting the manuscript. GP and FD contributed to study conception and interpretation of results and revised the manuscript critically for important intellectual content. PS, OL, and JN contributed to data acquisition and revised the manuscript critically for important intellectual content. MS contributed to study conception and revised the manuscript critically for important intellectual content. All authors read and approved the final manuscript.

## Funding

This study was performed with the support of the Italian Cooperation Agency (AICS—Agenzia Italiana per la Cooperazione allo Sviluppo).

## Conflict of Interest

The authors declare that the research was conducted in the absence of any commercial or financial relationships that could be construed as a potential conflict of interest.

## Publisher's Note

All claims expressed in this article are solely those of the authors and do not necessarily represent those of their affiliated organizations, or those of the publisher, the editors and the reviewers. Any product that may be evaluated in this article, or claim that may be made by its manufacturer, is not guaranteed or endorsed by the publisher.
